# Safe injection, infusion and medication-vial practices at a tertiary care centre: a quality improvement initiative

**DOI:** 10.3205/dgkh000429

**Published:** 2023-01-27

**Authors:** Pragathi Kottapalli, Naveen Chander Reddy Podduturi, Ganta Aswini, Somisetty Jyothi, Admala Naveen

**Affiliations:** 1 Department of Hospital Infection Control, AIG Hospitals, Hyderabad, Telangana, India; 2 Department of Hospital Administration, AIG Hospitals, Hyderabad, Telangana, India

**Keywords:** safe injection and infusion practices, aseptic techniques, single syringe, single needle, single patient, multidose-vial policy, labelling of IV lines, safe disposal of sharps

## Abstract

**Introduction:**

There is a risk of transmission of viruses and microbial pathogens during routine health care procedures due to improper injection, infusion, and medication-vial practices. Unsafe practices lead to outbreaks of infection resulting in unacceptable and devastating events in patients. The present study was undertaken to assess the compliance of nurses with safe injection and infusion practices in our hospital and to identify staff education requirements in relation to the safe-injection and infusion practices policy.

**Methods:**

Baseline data were collected and high risk areas were identified on this basis, a quality improvement project was implemented by infection control team. FOCUS PDCA methodology was used to conduct the improvement process. The study was performed from March to September 2021. An audit checklist based on the CDC guidelines was used for monitoring compliance with safe injection and infusion practices.

**Results:**

Poor compliance with safe injection and infusion practices in few clinical areas at baseline. During the pre-intervention period, non-compliance was mainly seen with the following elements: aseptic technique (79%), rubber septum disinfected with alcohol (66%), labelling of all IV lines and medications with date and time (83%), compliance with multidose-vial policy (77%), use of multidose vials for single patient (84%), safe disposal of sharps (84%), using trays instead of clothing/pockets to carry medications (81%). There was significant improvement in compliance with the following elements of safe injection and infusion practices in the post-intervention period: aseptic technique (94%), rubber septum disinfected with alcohol (83%), compliance with multidose-vial policy (96%), use of multidose vials for single patient only (98%), safe disposal of sharps (96%).

**Conclusion:**

Adherence to safe injection and infusion practices is very important to prevent outbreaks of infection in health care settings.

## Zusammenfassung

**Einführung:**

Im Fall unsachgemäßer Injektion, Infusion und fehlerhaften Umgangs mit Medikamenten besteht das Risiko der Übertragung von Viren und Mikroorganismen. Fehlerhafte Praktiken können zum Ausbruch von Infektionen mit drastischen Folgen für die Patienten führen. Die vorliegende Studie wurde durchgeführt, um die Einhaltung sicherer Injektions- und Infusionspraktiken in unserem Krankenhaus durch das Pflegepersonal zu bewerten und den Schulungsbedarf des Personals zu ermitteln.

**Methoden:**

Die Analyse der Ausgangssituation ergab eine schlechte Einhaltung sicherer Injektions- und Infusionspraktiken in einigen klinischen Bereichen. In den identifizierten Bereichen mit hohem Risiko wurde vom Infektionskontrollteam ein Qualitätsverbesserungsprojekt etabliert. Zur Durchführung wurde die FOCUS PDCA-Methode verwendet. Die Studie erstreckte sich von März bis September 2021. Zur Überwachung der Einhaltung sicherer Injektions- und Infusionspraktiken wurde eine Audit-Checkliste auf der Grundlage der CDC-Richtlinien verwendet.

**Ergebnisse:**

Während der Zeit vor der Intervention wurde die Nichteinhaltung hauptsächlich in Bezug auf folgende Elemente festgestellt: Aseptische Technik (79%), mit Alkohol desinfiziertes Gummiseptum (66%), Kennzeichnung aller Infusionsleitungen und Medikamente mit Datum und Uhrzeit (83%), Einhaltung der Richtlinie für Mehrdosisfläschchen (77%), Verwendung von Mehrdosisfläschchen für einen einzelnen Patienten (84%), sichere Entsorgung von scharfen Gegenständen (84%), Tragen von Medikamenten in Kleidung/Taschen anstatt auf dem Tablett (81%). Es gab eine signifikante Verbesserung bei der Einhaltung der folgenden Elemente sicherer Injektions- und Infusionspraktiken nach der Intervention: Aseptische Technik (94%), mit Alkohol desinfiziertes Gummiseptum (83%), Einhaltung der Richtlinie für Mehrdosis-Durchstechflaschen (96%), Verwendung von Mehrdosis-Durchstechflaschen für einen einzelnen Patienten (98%), sichere Entsorgung scharfer Gegenstände (96%).

**Schlussfolgerung:**

Die Einhaltung sicherer Injektions- und Infusionspraktiken ist unerlässlich, um Ausbrüche nosokomialer Infektionen zu verhindern.

## Introduction

The World Health Organization (WHO) defines a safe injection as one which does not harm the recipient, does not expose the provider to avoidable risk, and does not result in waste that is dangerous for the community. It is expected that this evidence-based policy guidance will additionally contribute to preventing the re-use of syringes on patients, thus decreasing the rate of needle-stick injuries in health-care workers (HCWs) related to injection procedures. They are part of standard precautions which help in the prevention of transmission of infections due to unsafe injections [1].

Injectable medicines are commonly used in health-care settings for the diagnosis, treatment and prevention of various illnesses. The Centers for Disease Control and Prevention (CDC) evidence-based Standard Precautions guideline on safe administration includes adherence to practices such as

1. not administering medications from the same syringe to more than one patient,
2. not entering a vial with a used syringe or needle,
3. administering medications from single-dose vials to multiple patients,
4. maintaining aseptic technique at all times,
5. disposing properly of used injection equipment,
6. not using bags of intravenous solution as a common source of supply for more than one patient,
7. not keeping multi-dose vials in the immediate patient treatment area [2], [3].

Globally, in the year 2000, approximately 20 million new hepatitis B virus (HBV) infections, 2 million new hepatitis C virus (HCV) infections, and 250,000 new human immunodeficiency virus (HIV) infections [2] were due to unsafe injection practices.

In developing countries, the WHO has estimated that about 16 billion injections are administered each year [4]. The estimated number of injections per person per year is 3.4 (range 1.7–11.3) and the proportion of unsafe injections is 39% (range 1.2–75%) [5]. In some areas of South East Asia including India, the WHO South East Asian (including India), the estimate for unsafe injection is greater than 75%. It has been estimated that in India, around three billion injections are administered annually, with 1.89 billion of them being unsafe [6].

There is risk of transmission of blood-borne viruses and microbial pathogens to patients during routine health care procedures due to improper injection, infusion and medication-vial practices. These unsafe practices are unacceptable and cause devastating events in patients. 

With this background, this study was conducted to assess the compliance of nurses with hospital policy on safe injection and infusion practices and identify staff education requirements in relation to the policy.

## Materials and methods

### Study design

This is an observational study.

### Study setting

The study was conducted at a tertiary care hospital in Hyderabad, Telangana, India, from March 2021 to September 2021. This hospital is an 800-bed multispecialty facility, accredited by NABH and JCI (Joint Commission International) and has comprehensive institutional policies for infection prevention and control as well as antimicrobial stewardship. The setting where the study was conducted included nursing staff of intensive care units, emergency departments, and inpatient wards.

The process flow chart showed that protocols on safe injection and infusion practices were made available at respective nursing stations. Staff were trained in these by the infection control team, but there were gaps in implementation of policy by nursing staff in all clinical areas.

**Figure 1:**
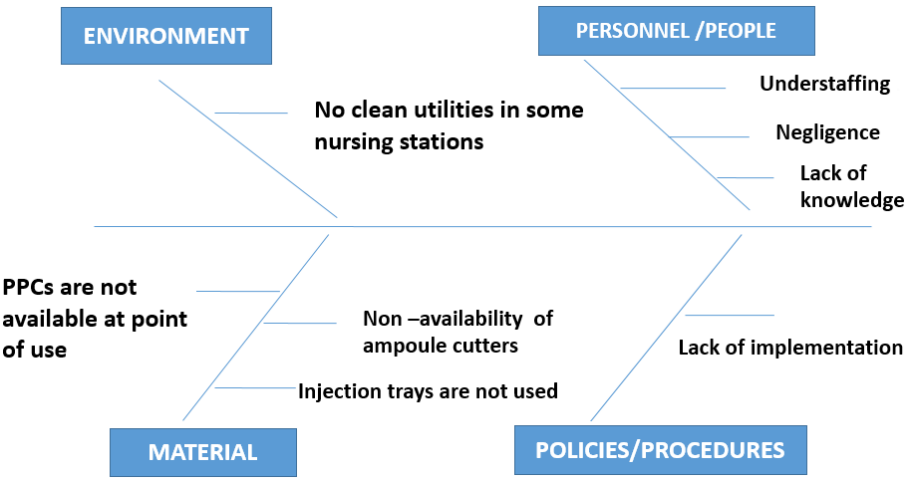
Fishbone analysis of problem

A fishbone analysis (Figure 1 (Fig. 1)) of the problem showed that there were no clean utilities in a few nursing stations for medication preparation. Hence, the nurses were preparing the medications at bedside. Some of the reasons for non-compliance were lack of knowledge, shortage of personnel and negligence. Inappropriate disposal of waste was due to non-availability of ampoule cutters and lack of puncture-proof containers at the point of use.

The quality improvement team decided to instruct the staff on the importance of safe injection and infusion practices, which is one component of standard precautions. It was planned to identify areas where clean utilities were not available.

### Phases of the study

The study included phase 1, the pre-intervention phase from March to May 2021, and phase 2, the interventional phase from April to August 2021.

**Figure 2:**
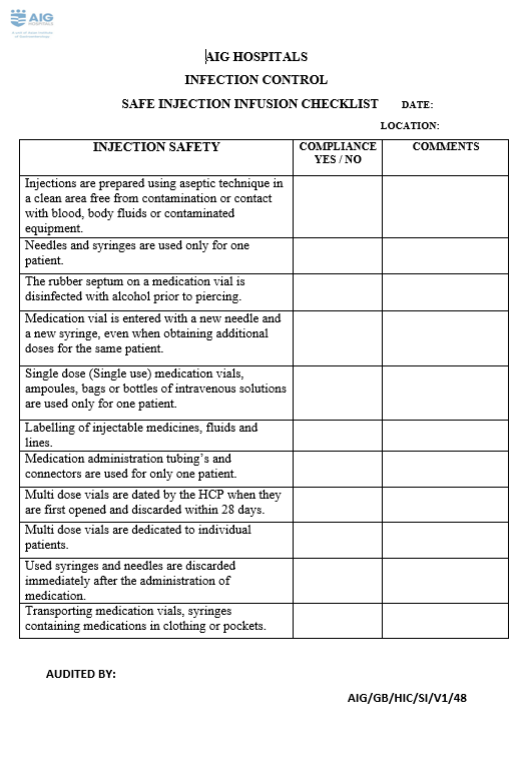
Revised injection safety assessment tool (Tool for data collection).xx in the 7^th^ cell, left column, change “Medication administration tubing’s” to “Medication administration: tubings”; in the last cell, left column, change “Transporting” to “Do not transport”

We performed the following activities during phase 1:

- Sample size and justification: Sample size was calculated as 152 by using a sample size calculator. This means 152 or more measurements/surveys are needed to have a confidence level of 95% that the real value is within ±5% of the measured or surveyed value.
- Sample selection method: During the study period, multiple structured observations were carried out by the infection control nurses and link nurses, who visited the clinical departments of the hospital to observe safe injection and infusion practices of the nurses by using a checklist to obtain accurate information and review compliance with safe injection practices.
- Tools of study: A pre-designed data collection checklist adopted and modified from the CDC guideline of the revised injection safety assessment tool was used as a tool in this study (Figure 2 (Fig. 2)). Safe injection and infusion practices is one of our hospital infection control quality indicators, and data is collected by infection control nurses using an audit checklist. Non-compliance with any of the components in the checklist was taken as 100% non-compliance. The data is compiled, analyzed and presented at the hospital infection control committee meeting every month (Table 1 (Tab. 1)). Investigators visited the study settings and observed the procedure of safe injection and infusion practices during their daily surveillance. These visits were not announced ahead of time.
- Baseline measures: The data compiled revealed that the compliance with safe injection and infusion practices was very poor. Root cause analysis was done and various factors for non-compliance were identified in the current process of safe injection and infusion practices.

During phase 2, the following activities were realized:

- April 2021: I. A meeting was held by the infection control team with the nursing department, and feedback on safe injection and infusion practices was shared. II. Interventional strategies were planned and a quality improvement plan (QIP) on safe injection and infusion practices was agreed upon by both teams. III. The QIP team was organised. IV. A quality improvement team was formed involving the infection control officer, infection control nurses and link nurses from each nursing station.
- May 2021: I. This team followed FOCUS PDCA methodology to conduct the improvement process. II. The quality improvement team conducted brainstorming sessions and used process flowcharts and Fishbone analyses to analyse the problem of unsafe injection and infusion practices.
- June 2021: I. Risk areas with highest percentage of non-compliance were identified. II. Training of nursing staff on safe injection and infusion practices was planned.
- July to August 2021: The goals were to: I. make small disposable puncture-proof containers (PPCs) available in all clinical areas; II. identify areas without clean utilities; III. inform hospital administration about the requirement; IV. conduct re-audit in all clinical areas in September 2021; V. review the strategies and add further interventions if there was no improvement; VI. sustain the results in case of improvement.
- September 2021: I. A re-audit was carried out by infection control nurses. II. Data were compiled and results were analysed. III. There was significant improvement of practices in the identified high-risk areas along with other areas. IV. Ongoing training of nursing staff was planned.V. Link nurses were identified from all wards/OTs/ICUs. VI. Each link nurse was to train nursing staff in their respective areas on safe injection and infusion practices. VII. Continuous monitoring of safe injection and infusion practices was performed by infection control nurses. VII. Feedback on the audit was given to the infection control committee and nursing department for improvement. VIII. Provisions for clean utilities were made in all nursing stations. IX. To sustain the results of improvement, compliance with safe injection and infusion practices was made one of the monthly infection control quality indicators.

## Results

During analysis of the compiled data, non-compliance with even one element in the audit checklist was considered as 100% non-compliance, because unsafe practices are unacceptable and may cause outbreaks leading to devastating events in patients.

The data compiled during the pre-intervention phase revealed some high-risk areas with poor compliance with safe injection and infusion practices (Table 2 (Tab. 2)).

**Figure 3:**
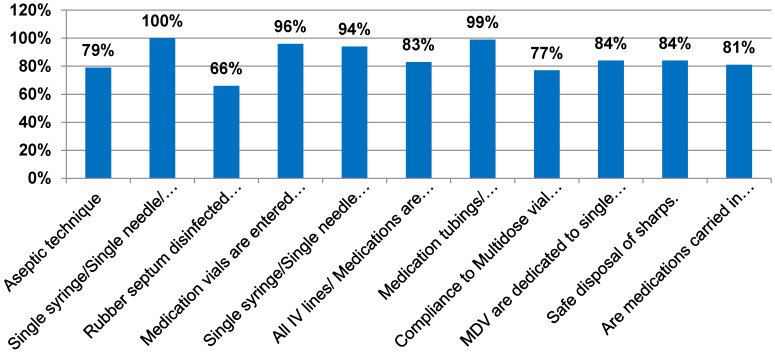
Compliance percentage with various elements of safe injection and infusion practices

Compliance percentage with various elements of safe injection and infusion practices was calculated. Non-compliance was mainly seen with the following elements: aseptic technique (79%), rubber septum disinfected with alcohol (66%), labelling of all IV lines and medications with date and time (83%), compliance with multidose-vial policy (77%), use of multidose vials for single patient (84%), safe disposal of sharps (84%), using trays instead of carrying medications in clothing/pockets (81%) (Figure 3 (Fig. 3)).

An improvement plan was made and actions were executed within the time frames given in Table 3 (Tab. 3).

Re-auditing was conducted by infection control nurses following implementation of interventional strategies. Data analysis showed a significant decrease in non-compliance with safe injection and infusion practices (Table 4 (Tab. 4)).

**Figure 4:**
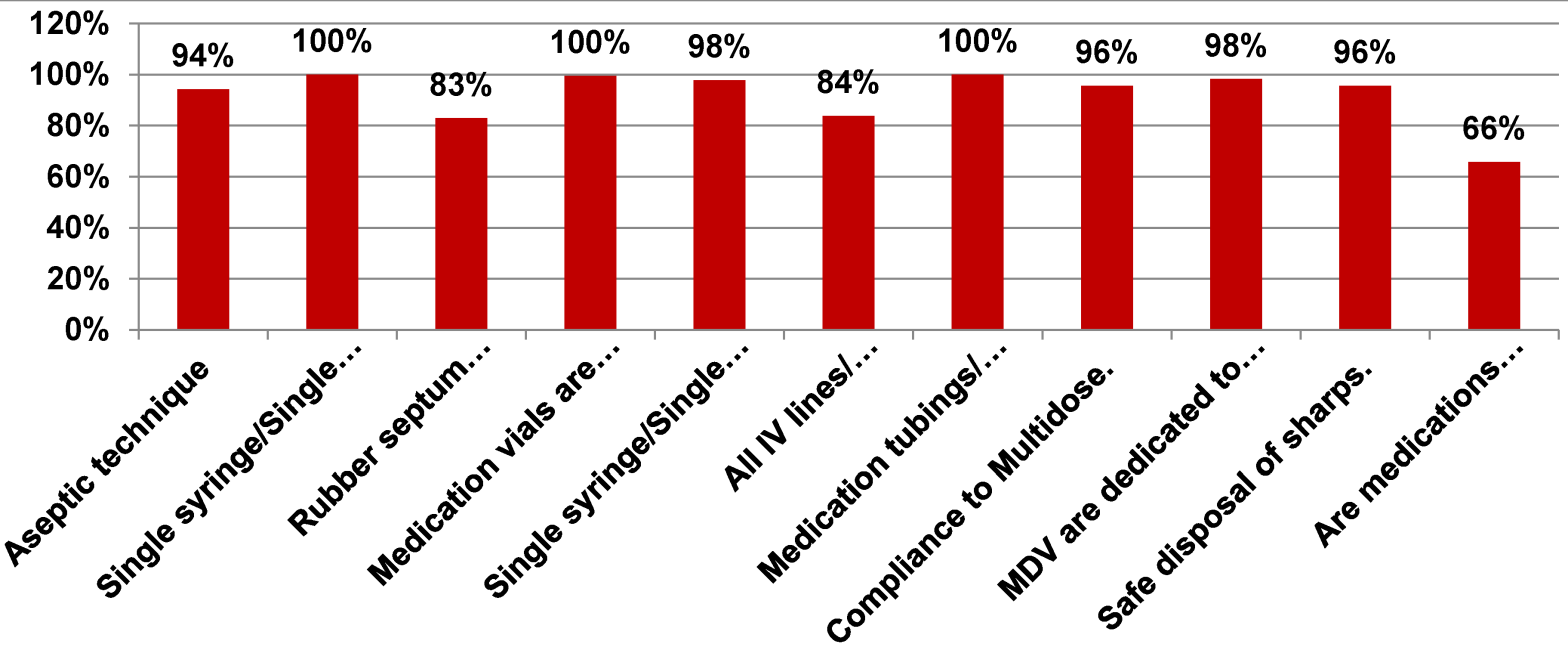
Compliance with various elements of safe injection and infusion practices

Significant improvement was found in compliance with the following elements of safe injection and infusion practices in the post-intervention period: aseptic technique (94%), rubber septum disinfected with alcohol (83%), compliance with multidose-vial policy (96%), use of multidose vials for single patient (98%), safe disposal of sharps (96%) (Figure 4 (Fig. 4)).

There was only marginal improvement in labelling of IV lines and medications. The quality improvement project was presented at the infection control committee meeting for approval. The quality improvement team achieved its aim and is sustaining it to date.

## Discussion

With this quality improvement project, we identified all clinical areas requiring clean utilities, which is a key component for implementing safe injection and infusion practices. In a study by Rajneesh et al. [7], similar unsafe injection practices were observed, such as not preparing injections on a clean workable tray (35%), not removing needles from the cap of multidose vials (40%), recapping needles (4%), not following hand hygiene (33%) and not using clean barriers (87.5%) to protect fingers while breaking glass ampoules in the study hospital.

Many nursing staff had suffered needlestick injuries due to poor compliance with safe disposal of sharps and non-availability of PPCs at the point use. The incidence of needlestick injuries decreased following training of staff on safe disposal of sharps [8], [9], [10].

The importance of adherence to the aseptic technique was recognised by the nurses after taking up this project [11], [12]. A study by Paul et al. [12] at a tertiary care hospital in West Bengal showed similar results. Only 12.5% of study subjects performed hand hygiene before giving injections and only 3.7% of them used gloves during injection. Our compliance rate was higher compared to Paul’s study and similar to studies in Egypt, Nepal and West Bengal [13], [14], [15].

Our quality improvement project allowed the team to improve patient care in all clinical areas, including very busy areas such as the emergency department and intensive care units. The success was also dependent on good communication and coordination between the infection control team and the nursing department. The team plans to carry this process forward by continuous training and monitoring staff practices.

## Conclusions

Adherence to safe injection and infusion practices is very important to prevent outbreaks of infection in health-care settings. This quality improvement project helped us identify gaps in our current practices and make significant improvement for better patient care. Continuous health-care professional training on infection control and safe injection and infusion practices should be encouraged. Frequent infection control auditing is mandatory to ensure compliance with safe injection and infusion practices.

## Notes

### Competing interests

The authors declare that they have no competing interests.

### Acknowledgement

We acknowledge the support of the hospital administration and all the nurses who actively participated in the implementation of standardised safe injection and infusion practices for safe and effective patient care.

## Figures and Tables

**Table 1:**
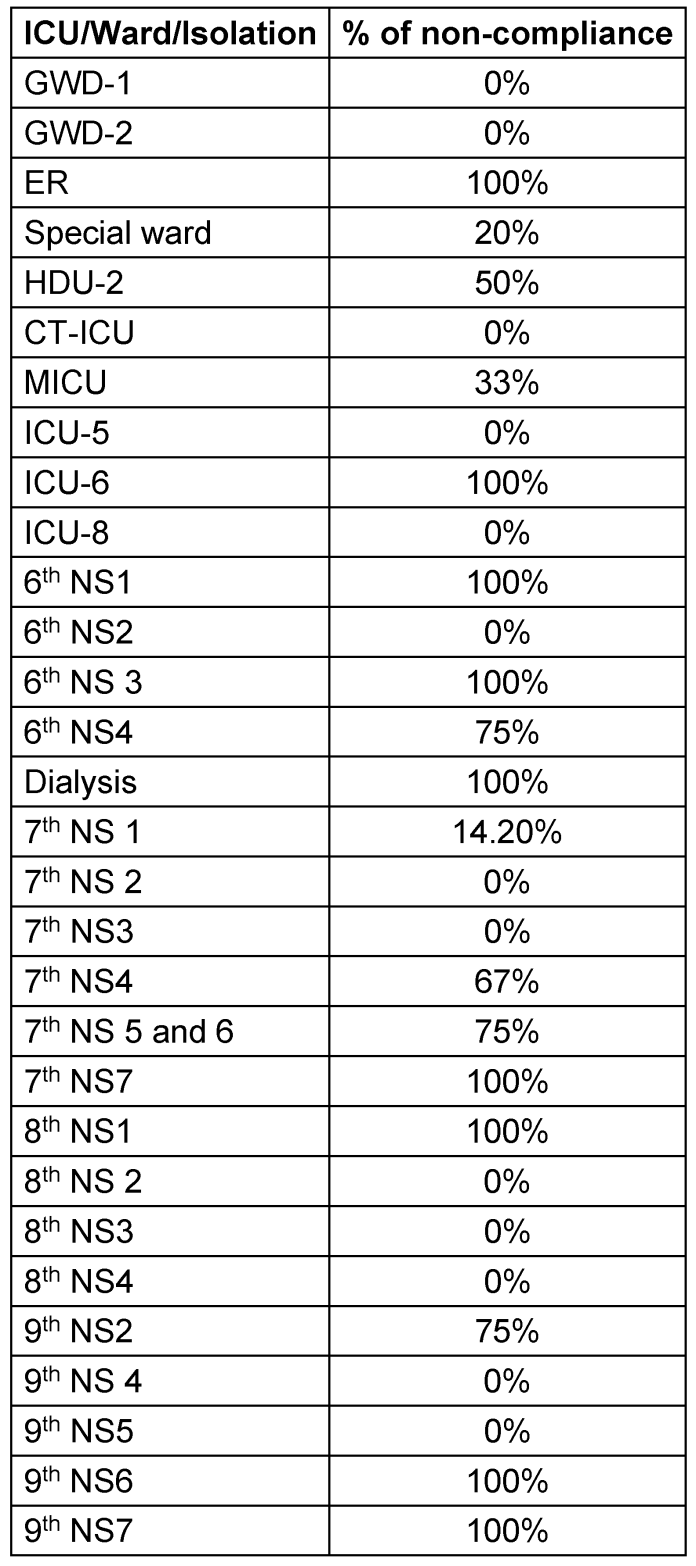
Pre-intervention non-compliance (%) in different clinical areas

**Table 2:**
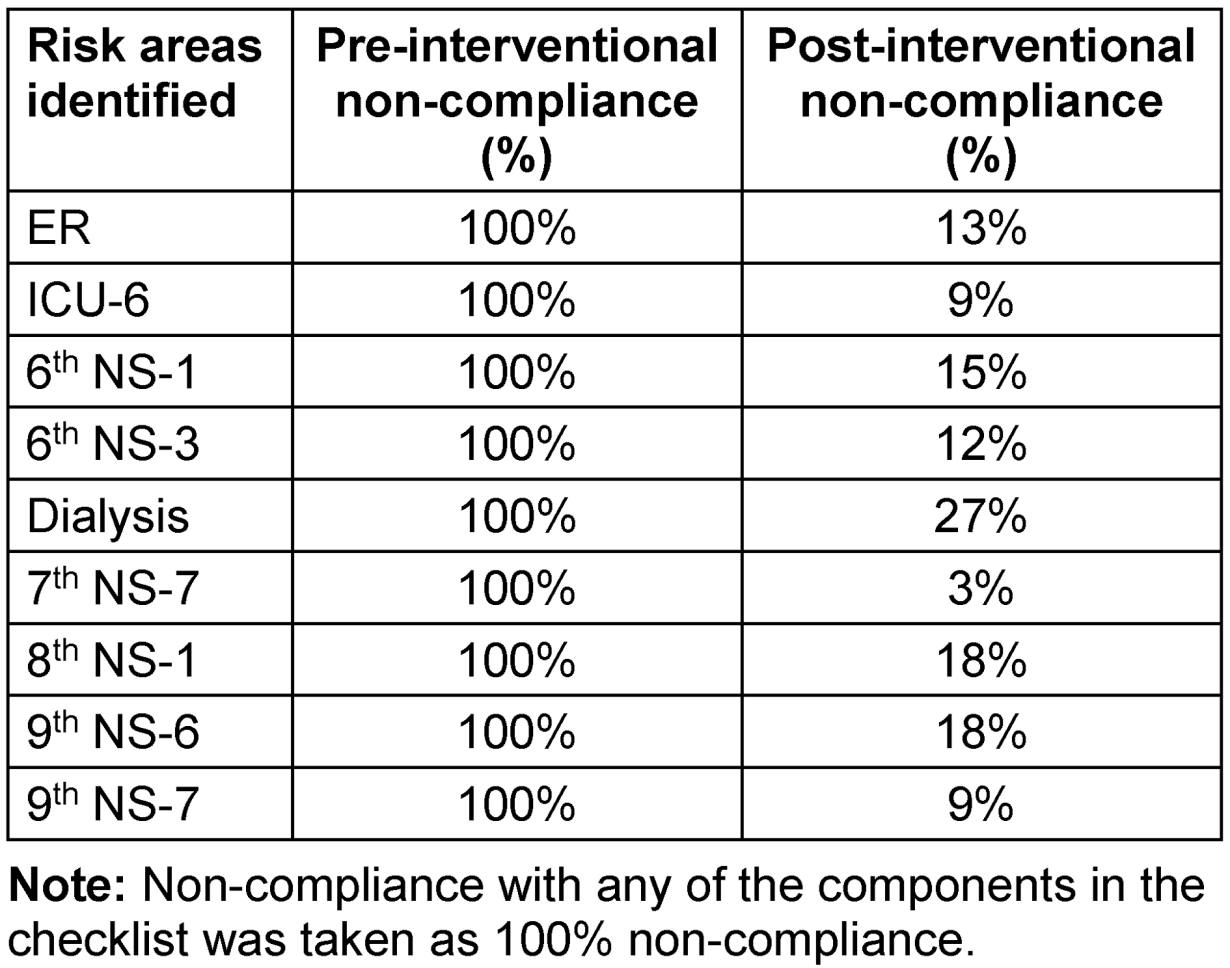
Pre- and post-interventional non-compliance rates in risk areas

**Table 3:**
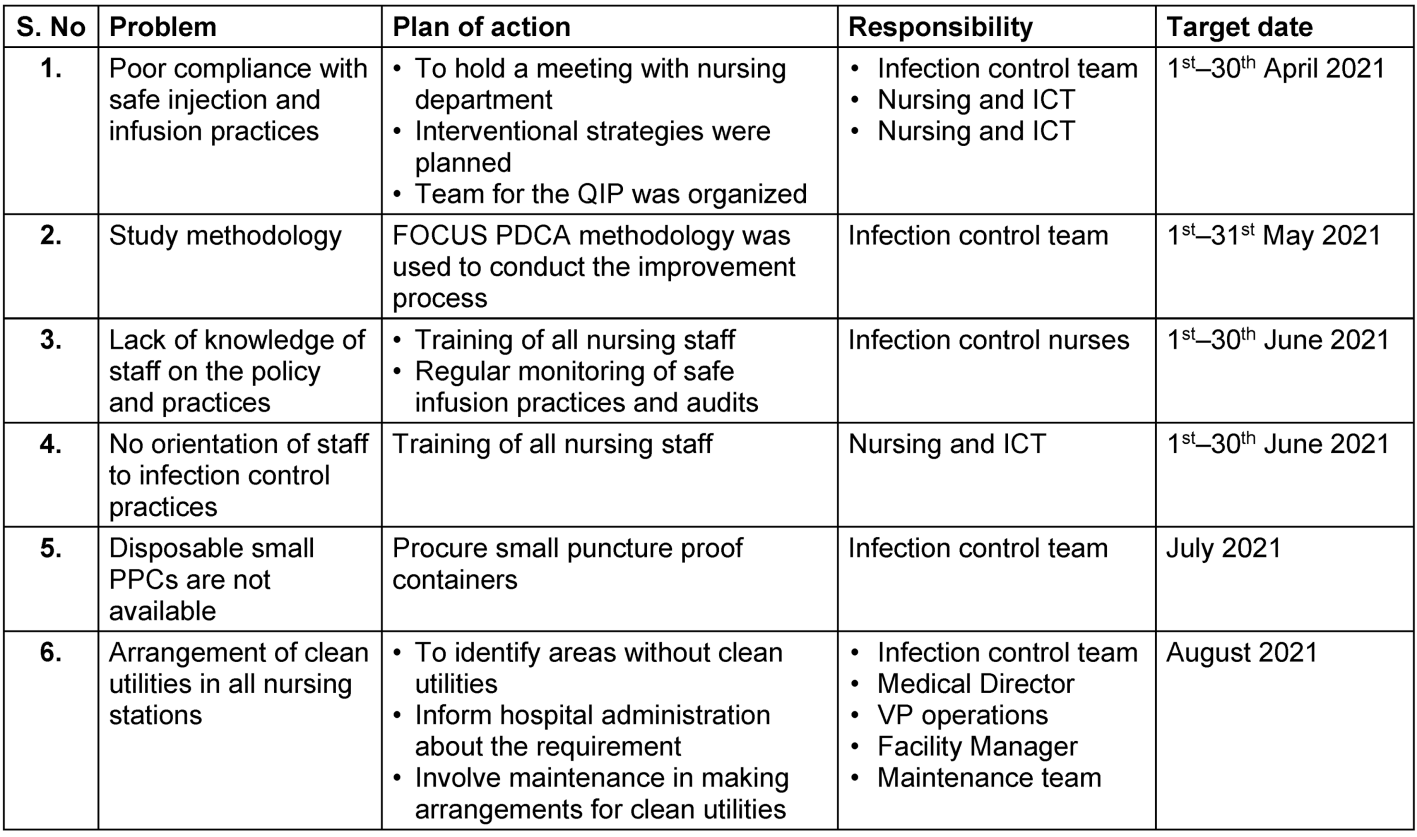
Plan of improvement

**Table 4:**
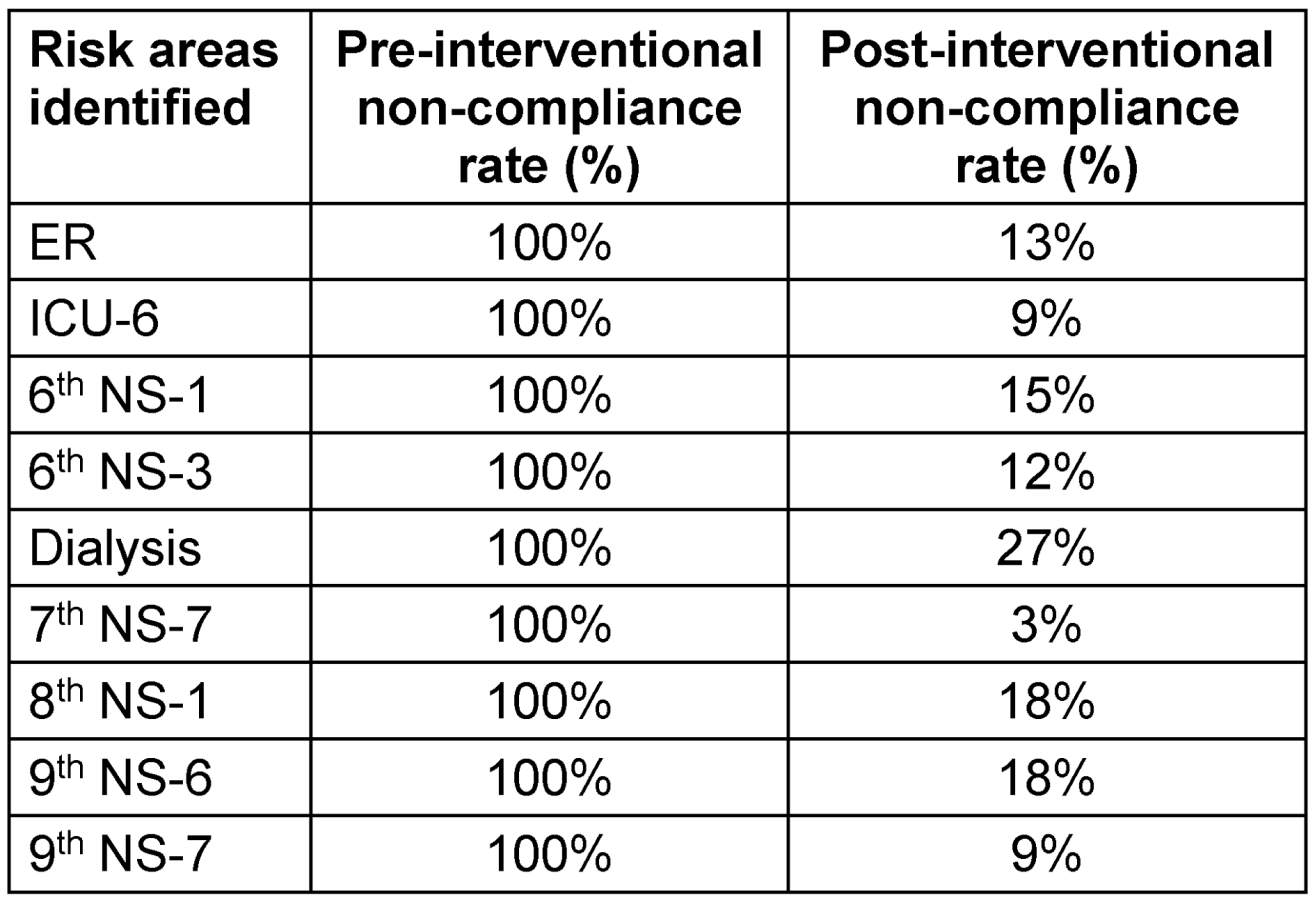
Pre- and post-interventional non-compliance rates
